# Concordance between child and parent-proxy report on a new self-report tool of vision related quality of life for children with JIA-associated uveitis – “Effects of Youngsters’ Eyesight on QOL -EYE-Q”

**DOI:** 10.1186/1546-0096-10-S1-A43

**Published:** 2012-07-13

**Authors:** Sheila T Angeles-Han, Sampath Prahalad, Lori Ponder, Kerrie Fields, Rachel Robb, Marla Shainberg, Larry B Vogler, Phoebe Lenhart, Amy Hutchinson, Sunil K Srivastava, Scott R Lambert, Carolyn Drews-Botsch

**Affiliations:** 1 Children's Healthcare of Atlanta, Atlanta, GA, USA; 2 Cleveland Clinic Foundation, Cleveland, OH, USA; 3 Emory Children's Center, Atlanta, GA, USA; 4 Emory University, Atlanta, GA, USA; 5 Emory University School of Medicine, Atlanta, GA, USA

## Purpose

Quality of life (QOL) studies in JIA-associated uveitis (JIA-U) focus on the ocular exam and global measures of QOL. However, these assessments may not accurately measure the impact of visual impairment (VI) on a child’s daily activities. Hence, a child-centered approach is crucial in this assessment. We report on the validity and reliability of a new self-report tool to measure vision related QOL (VRQOL) in children 8-18 years of age, “Effects of Youngsters’ Eyesight on QOL (EYE-Q)”.

## Methods

We recruited 120 children, 8-18 years of age, in a pediatric eye clinic. Best corrected visual acuity (VA) was measured using the Snellen chart. Patient-based questionnaires were administered– EYE-Q to measure VRQOL, and Pediatric Quality of Life Inventory (PedsQL) to determine overall QOL. Patients rated their eyesight as excellent (1), good (2), fair (3), poor (4), very poor (5) or completely blind (6). Parents and physicians assessed vision severity and scored how much the child’s life was affected by their vision from 1-10 (1 - vision does not affect function in daily activities, and 10 - vision affects function in daily activities).

## Results

Of 120 children, 48% were female, 61% were white, 46.7% had normal vision (both eyes with VA better than 20/40), 54.4% had VI (any eye with VA of 20/40 or worse) and 20.8% required the use of at least one visual aid. Mean age was 11.3 (SD 2.7). Mean scores of the measures in children with VI are shown in Table 1.

**Table 1:** Mean scores (SD) of measures in children with vision impairment (N=64)

	Vision impairment§
Measures	
Eye-Q^1^ (range of scores 0-4)^+^	3.22 (0.74)
PedsQL^2^ Total (0-100)^+^	76.0 (16.5)
Patient vision assessment (1-6)^++^	2.3 (1.2)
Patient vision assessment (1-10)^++^	5.6 (2.9)
Physician vision assessment (1-10)^++^	4.0 (2.5)

§ Vision impairment – any eye with a VA of 20/40 or worse

^1^Effects of youngsters' eyesight on QOL; ^2^Pediatric quality of life inventory score

^+^Higher scores indicate better quality of life, ^++^Higher values indicate worse vision severity

Patient vision assessment had a moderate association with EYE-Q (0.489); mild associations with VI (r = 0.266), VA (r = 0.340), physician vision assessment (r = 0.315), and need for visual aids (r = -0.281); and no association with PedsQL, laterality of VI, and parent vision assessment (Table 2). Parent vision assessment had mild associations with VI (r = 0.280), VA (r = 0.239), EYE-Q (r = -0.297), need for aids (r = -0.281), and physician assessment (r = 0.384); and no association with Peds QL and laterality of VI. Patient vision assessment had stronger associations with the EYE-Q and VA compared to parent vision assessment.

**Table 2:** Correlations (r) between patient and parent vision assessments and measures of vision impairment§

	Patient vision assessment (1-6)^+++^	P value	Parent vision assessment (1-10)^+++^	P value
Vision impairment§	0.266	0.003**	0.280	0.002**
Eye-Q^1^ (range of scores 0-4)^+^	-0.489	<0.001*	-0.297	0.001**
PedsQL^2^ Total (0-100)^+^	-0.064	0.488	-0.053	0.569
Logmar far VA^3^, better eye (-0.30-3.00)^++^	0.340	<0.001*	0.239	0.009**
Need for visual aids	-0.281	0.002**	-0.281	0.002**
Laterality of disease	0.108	0.238	0.143	0.121
Patient vision assessment (1-10)^+++^	0.166	0.072		
Physician vision assessment (1-10)^+++^	0.315	<0.001*	0.384	<0.001*

^1^Effects of youngsters' eyesight on QOL; ^2^Pediatric quality of life inventory; ^3^Visual acuity

*<0.001, **<0.05 using person's coefficient

§ Vision impairment – any eye with a VA of 20/40 or worse

^++^Lower values indicate better vision and VA

^+++^Higher values indicate worse vision severity

## Conclusion

VI can have a significant impact on the VRQOL of children with ocular disease. Our study demonstrates that child-reported VRQOL and vision assessment has good validity but can differ from the parent’s assessment. Both parent and child reports may have valid contributions in the assessment of VRQOL and visual outcome. Hence, the child’s perspective should be included in QOL studies in JIA-U. A vision-specific self-report instrument like the EYE-Q may be an important tool in the assessment of VRQOL in JIA-U, may complement current measures of VI, and may be more valid than measures of overall QOL alone.

## Disclosure

Sheila T. Angeles-Han: None; Sampath Prahalad: None; Lori Ponder: None; Kerrie Fields: None; Rachel Robb: None; Marla Shainberg: None; Larry B. Vogler: None; Phoebe Lenhart: None; Amy Hutchinson: None; Sunil K. Srivastava: Bausch and Lomb, 9; Scott R. Lambert: None; Carolyn Drews-Botsch: None.

